# Mechanics of walking and running up and downhill: A joint-level perspective to guide design of lower-limb exoskeletons

**DOI:** 10.1371/journal.pone.0231996

**Published:** 2020-08-28

**Authors:** Richard W. Nuckols, Kota Z. Takahashi, Dominic J. Farris, Sarai Mizrachi, Raziel Riemer, Gregory S. Sawicki

**Affiliations:** 1 School of Engineering and Applied Sciences, Harvard University and Wyss Institute, Cambridge, Massachusetts, United States of America; 2 Department of Biomechanics, University of Nebraska at Omaha, Omaha, Nebraska, United States of America; 3 Department of Sport and Health Sciences, University of Exeter, St Luke's Campus, Exeter, United Kingdom; 4 Department of Industrial Engineering and Management, Ben-Gurion University of the Negev, Beer-Sheva, Israel; 5 School of Mechanical Engineering and Biological Sciences, Georgia Institute of Technology, Atlanta, Georgia, United States of America; University of Massachusetts Lowell, UNITED STATES

## Abstract

Lower-limb wearable robotic devices can improve clinical gait and reduce energetic demand in healthy populations. To help enable real-world use, we sought to examine how assistance should be applied in variable gait conditions and suggest an approach derived from knowledge of human locomotion mechanics to establish a ‘roadmap’ for wearable robot design. We characterized the changes in joint mechanics during walking and running across a range of incline/decline grades and then provide an analysis that informs the development of lower-limb exoskeletons capable of operating across a range of mechanical demands. We hypothesized that the distribution of limb-joint positive mechanical power would shift to the hip for incline walking and running and that the distribution of limb-joint negative mechanical power would shift to the knee for decline walking and running. Eight subjects (6M,2F) completed five walking (1.25 m s^-1^) trials at -8.53°, -5.71°, 0°, 5.71°, and 8.53° grade and five running (2.25 m s^-1^) trials at -5.71°, -2.86°, 0°, 2.86°, and 5.71° grade on a treadmill. We calculated time-varying joint moment and power output for the ankle, knee, and hip. For each gait, we examined how individual limb-joints contributed to total limb positive, negative and net power across grades. For both walking and running, changes in grade caused a redistribution of joint mechanical power generation and absorption. From level to incline walking, the ankle’s contribution to limb positive power decreased from 44% on the level to 28% at 8.53° uphill grade (*p* < 0.0001) while the hip’s contribution increased from 27% to 52% (*p* < 0.0001). In running, regardless of the surface gradient, the ankle was consistently the dominant source of lower-limb positive mechanical power (47–55%). In the context of our results, we outline three distinct use-modes that could be emphasized in future lower-limb exoskeleton designs 1) Energy injection: adding positive work into the gait cycle, 2) Energy extraction: removing negative work from the gait cycle, and 3) Energy transfer: extracting energy in one gait phase and then injecting it in another phase (*i*.*e*., regenerative braking).

## Introduction

Lower-limb robotic exoskeletons can apply assistive torque to reduce the metabolic energy used by biological muscles to produce the force and work for locomotion [1]. A majority of these successful exoskeletons have focused on providing assistance at the ankle within a laboratory setting [2–10]. More recently, devices have begun to move outside of laboratory confinement. Fully-autonomous, portable devices have been demonstrated to reduce metabolic cost by 10% during walking [4], by 14.9% with additional load [11], and by 14.6% during running [12]. To receive benefit from a device, users must learn to interact effectively with their wearable robot [8, 13].

Researchers have dedicated significant time and effort to understanding the interaction between exoskeleton control strategies and the physiological response of the human user. The high-level method for generating control commands [14, 15]; the shape, the timing and magnitude of the torque assistance profile [16–18]; and the lower-limb joint where assistance is targeted [18–21] can all influence how well the user responds. To date, most exoskeleton research studies have focused on optimizing controllers for a single gait at a fixed speed on level ground. Exhaustive parameter sweeps and human-in-the loop optimization have been very effective for determining torque profiles on an individual basis [7, 8, 22, 23], but discovering an optimal policy can take many hours. Furthermore, it is unknown how well control policies established for one condition can be generalized for the diverse gait conditions expected in the real-world.

As exoskeletons become increasingly mobile, a clear problem arises: How can engineers deliver systems that can assist in natural environments where locomotion involves adjusting speed, changing gait from walk to run, and moving uphill or downhill? Few exoskeleton studies have focused on incline/decline walking [5, 24] or compared assistance strategies across speeds [10] in which mechanistic explanations for performance outcomes were provided. Injection of positive power has been shown to be a promising approach for achieving metabolic cost reduction [25]; however, whether this approach is effective across all leg joints or if it is effective across different grades or gaits is unknown. We suggest that a bio-inspired mechanistic understanding of how people move and exchange energy between their lower-limb joints and the external environment is crucial for successful designs that make exoskeletons truly effective in real-world conditions. These insights may be directly incorporated into assistance profiles or may be used to seed optimization parameters.

This mechanistic approach has been previously applied to exoskeleton development and logically helps explain why the field has so heavily focused on the ankle as a target for assistance in level walking [3, 26, 27]. The ankle provides the majority of power on level ground [28] and disrupted ankle mechanics common in clinical populations make it a good target for assistance [29, 30]. Guidance from baseline human gait data has motivated a bioinspired approach to borrow ‘best’ concepts from the biological system to guide design of wearable devices. For example, our previous work to design and test a clutch-spring ankle ‘exo-tendon’ [23, 26, 27] was inspired by the efficient interaction between the triceps surae muscles and the Achilles tendon within the ankle plantarflexors during walking [31, 32].

The same mechanistic approach can be applied towards the development of exoskeletons in non-level gait. When moving on inclines and declines, fundamental physics shape the mechanical demands on the legs. Muscles must add or remove net mechanical energy lost or gained according to changes in height of the center of mass (COM) [33, 34] and numerous studies have contributed to our understanding of the dynamics of uphill and downhill gait at various speeds [35–45]. Joint level mechanical analyses through inverse dynamics have provided more detailed insight into the sources of mechanical energy generation/dissipation moving uphill/downhill, respectively. In general, the magnitude of hip extension moments increase during incline walking to inject net mechanical work; and magnitude of knee extension moments increase during decline walking to extract net mechanical work [36, 41, 42]. In incline running, the required increase in energy also results from a shift in net power output to the hip [35, 42]. Inverse dynamics analysis has also been used to evaluate the effect of aging on the joint kinematics and kinetics of uphill walking and reveals that older adults perform more hip work and less ankle work in both level ground and incline walking [38]. Other studies have demonstrated that individual joint dynamics can be used as a predictive tool for estimating the metabolic cost of walking, with 89% of the added metabolic cost of incline walking explained through changes in joint kinematics and kinetics [37].

**Fig 1:**
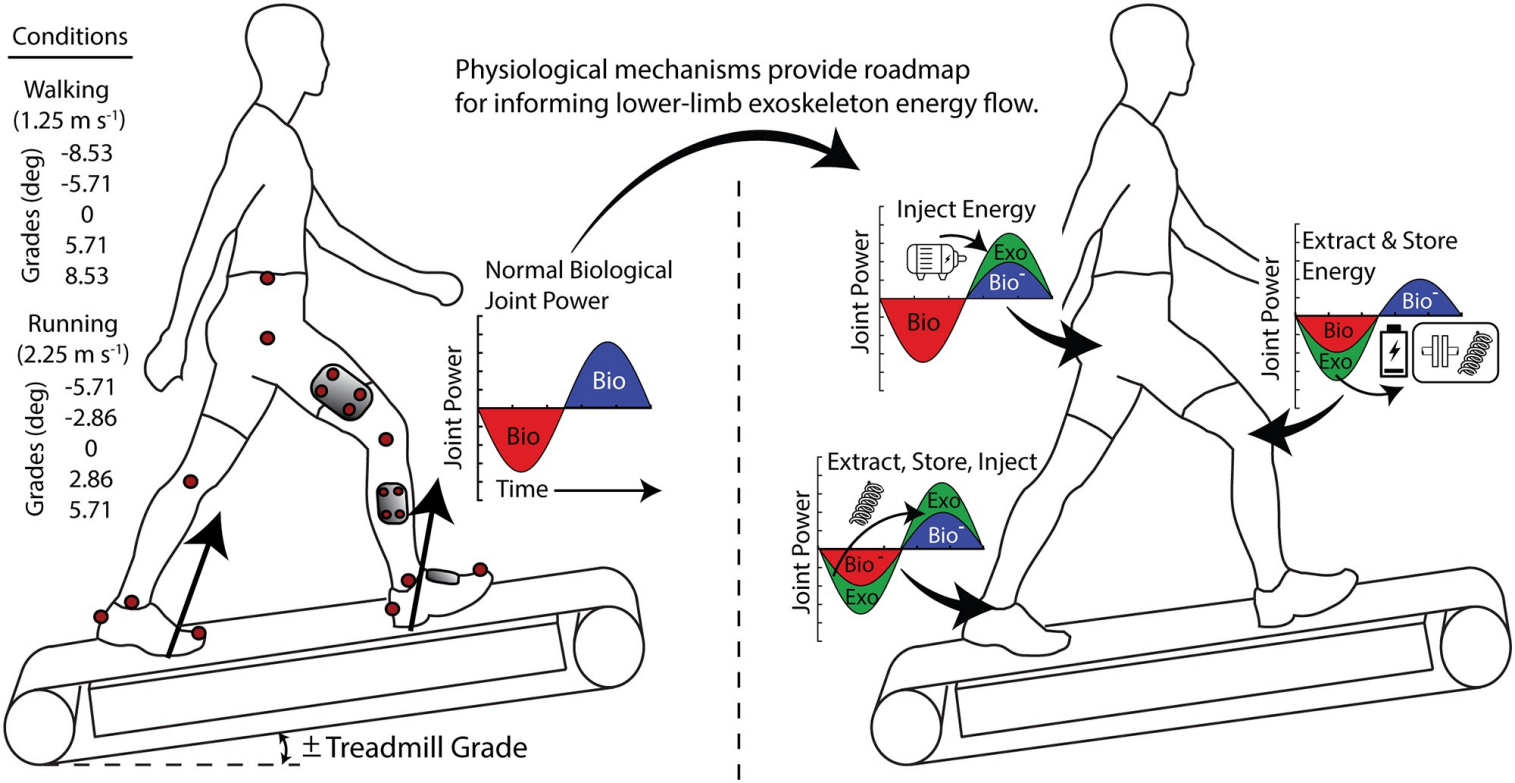
Schematic of experimental design and analysis. (A) Representation of gait conditions for characterizing changes in lower-limb mechanics during walking and running across incline and decline grades. (B) Example of energy cycle and potential mechanisms for how physiological mechanisms may provide a roadmap for informing lower-limb exoskeleton development.

The purpose of this study was to characterize changes in lower-limb joint mechanics during both walking and running across a range of incline/decline grades and then provide an analysis that informs lower-limb exoskeleton development (Fig 1). We hypothesized that the distribution of limb-joint positive mechanical power would shift to the hip for incline walking and running. For decline walking and running, we hypothesized that the distribution of limb-joint negative mechanical power would shift to the knee. In our analysis we sought to add an applied twist to current basic science understanding by focusing interpretation of the measured changes in human joint mechanics to guide the development of versatile exoskeleton systems with the ability to inject (net positive work), extract (net negative work) and transfer (net zero work) mechanical energy to meet variable mechanical demands of real-world environments.

## Methods

Eight adults (6M,2F, age: 23.38±4.10 yrs; mass 75.39±11.57 kg; height 177±0.07 cm) participated in the study. All subjects were healthy and gave written informed consent to participate in the study. The protocol and all testing were approved by the University of North Carolina at Chapel Hill Institutional Review Board.

Subjects completed five walking (1.25 m s^-1^) and five running (2.25 m s^-1^) trials over a range of incline and decline grades (Fig 1). Walking trials were at -8.53°, -5.71°, 0°, 5.71°, and 8.53° (-15%, -10%, 0%, 10%, and 15% grade) and running trials were at -5.71°, -2.86°, 0°, 2.86°, and 5.71° (-10%, -5%, 0%, 5%, and 10% grade). The grade for running was reduced due to the high demand on individuals at steep grade. The ranges provided an overlap at the -5.71°, 0°, and 5.71° grade for comparison between the two gaits. All trials were completed in the same day, and to minimize fatigue, subjects were given rest breaks in between trials. Experimental trials took place on a split belt instrumented treadmill with incline and belt velocity reversal functions (Bertec Inc., Columbus, OH, USA). Decline gait was obtained by inclining the treadmill and reversing the belt velocity. Walking and running trials each lasted 7 minutes. Walking and running trials were pseudorandomized, and once the treadmill incline was set, all conditions for that grade were completed.

Joint kinematic data were recorded using an eight-camera motion capture system (Vicon Inc., Oxford, UK, 120 Hz) to record the position of 22 reflective markers on the right lower limb and pelvis. Raw marker positions were filtered using a 2^nd^ order, low pass filter with a cut off frequency of 10 Hz. Segment tracking was performed by placing rigid plates containing clusters of 3–4 markers on the foot, shank, thigh, and pelvis. Calibration landmarks and relative location of tracking markers were identified through a standing trial that was performed at the beginning of the trials. The tracking markers were recorded during each trial and the orientation of the distal segment relative to the proximal segment was used to define the 3D joint angle. Ground reaction force (GRF) data were captured through the force plates embedded in the instrumented treadmill (Bertec Inc., Columbus, OH, USA, 960 Hz). GRF data were filtered with a 2^nd^ order low pass Butterworth filters with a cut off frequency of 35 Hz.

The GRF and the kinematic data from the individual limbs were used to perform an inverse dynamics analysis. We performed inverse dynamics at the joint level using commercially available software (Visual 3D, C-motion Inc., USA). Calculations of the time-varying moment and power were performed at the ankle, knee, and hip for a stride. A stride was defined from heel strike to heel strike of the right foot. We analyzed the entire stride due the importance of capturing the power that is performed by the hip and knee in the swing phase. Average positive and negative power (W kg^-1^) was calculated for each joint at each condition. Average positive power for each joint over the stride was calculated by integrating periods of only positive joint power with respect to time. This positive joint work (J kg^-1^) was then averaged across all of the strides. Average joint positive mechanical power was calculated by dividing the average joint positive work by the average stride time for the trial. The total limb average positive power was calculated by summing the average positive power at each joint (total = hip + knee + ankle). Next, each individual joint’s percent contribution to the total limb average positive power for the stride was calculated by dividing that joint’s average positive power by the total limb average positive power. The same process was followed to compute stride average negative power, where only the contribution of negative work at each joint was used. The average net power for each joint and for the limb was then calculated by summing the positive and negative average power values at each joint and for the limb.

For each gait (walk and run), we performed a repeated measures ANOVA (rANOVA, main effect: grade) to test the effect of grade on stride average joint power of the ankle, knee, and hip. (α = 0.05; JMP Pro, SAS, Cary, NC). In addition, for each gait (walk and run), we performed a repeated measures ANOVA (rANOVA main effect: joint) to evaluate the relative contribution of each joint at each grade. We applied a post-hoc Tukey HSD (HSD) test to evaluate for significance between conditions (either grade or joint). Finally, we performed matched pair t-test to evaluate the effect of gait (walk, run) on the stride average joint power contributions for similar grades (-5.71°, 0°, and 5.71°).

## Results

### Mechanical power in walking

#### Net power

**Fig 2:**
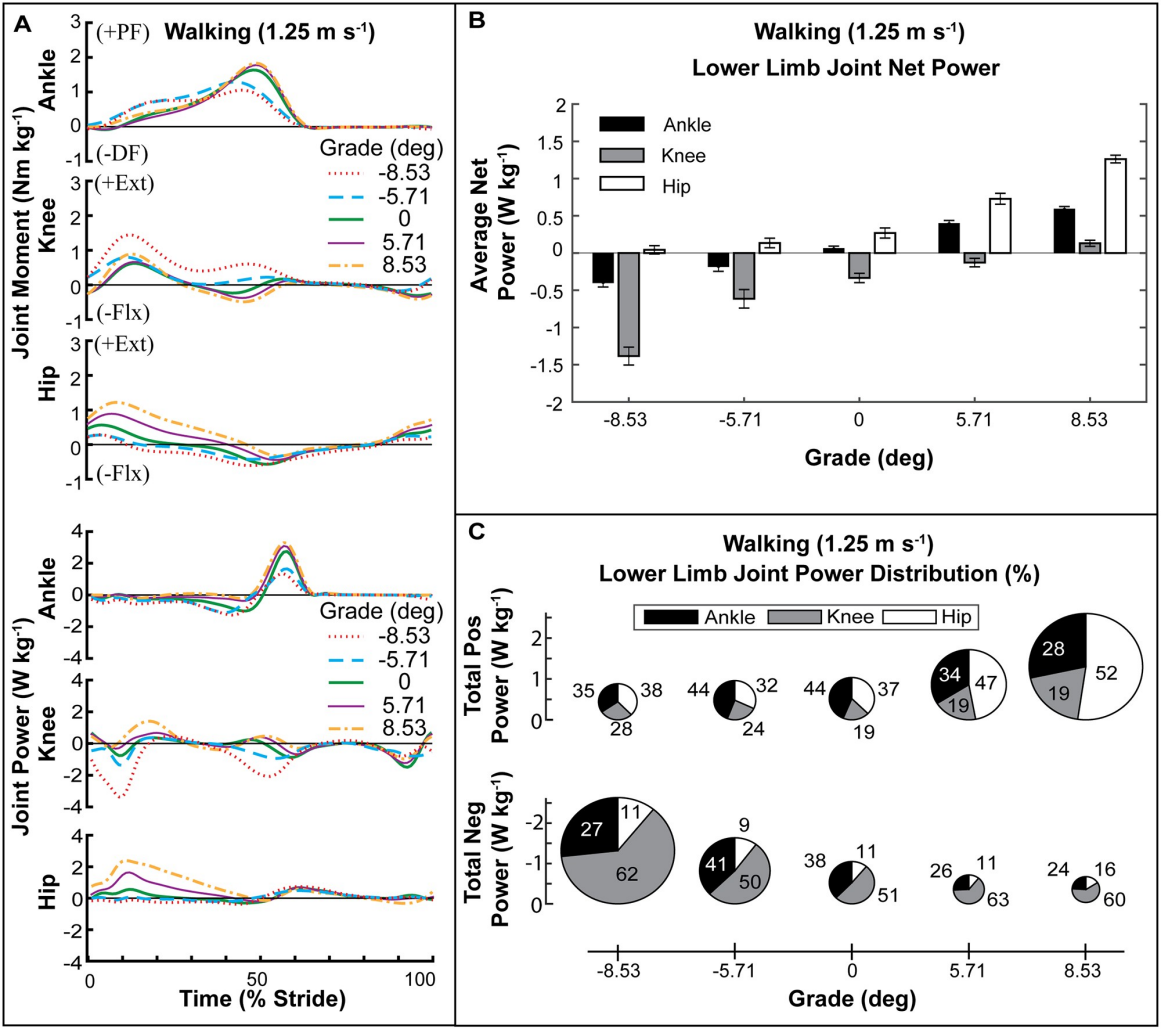
Lower-limb joint kinetics for walking at 1.25 m s^-1^ over a range of grades. **(A)** Joint moment and power over a stride for each grade. **(B)** Average net power of each joint across grade. **(C)** Percent distribution of average positive and negative lower-limb joint power. The diameter of each pie is normalized to the average positive power at level grade for walking (1.02 W kg^-1^).

The joint moments and powers were affected by grade (Fig 2). Average net mechanical power delivered by the ankle, knee, and hip all increased with grade (Fig 2B). The average net power of the ankle increased with grade (rANOVA, *p* < 0.0001), was negative for decline conditions, and positive for level ground and incline grades. The average net power of the knee was negative in all conditions except the +8.53° grade. The knee was the largest source of net negative power in all conditions, and the magnitude increased as grade decreased (rANOVA, *p* < 0.0001). The average net power of the hip was positive in all conditions and increased with grade (rANOVA, *p* < 0.0001). As incline increased, we observed an increased reliance on the hip for the required net positive power.

#### Positive power

The average positive power of the limb (ankle + knee + hip) increased with increasing grade (rANOVA, *p* < 0.0001) (Table 1; Fig 2C) from 1.02 W kg^-1^ at level to 1.70 W kg^-1^ (HSD, *p* < 0.0001) and 2.60 W kg^-1^ (HSD, *p* < 0.0001) at 5.71° and 8.53° grades respectively. Limb positive power was not significantly different from level at -5.71° and -8.53° grades respectively. The positive power of all three joints also increased individually with increased grade (rANOVA, *p* < 0.0001) (Table 1). However, the relative contribution of the ankle, knee, and hip to the total positive power of the limb changed with grade due to the unequal modulation of positive power at each joint for each grade (Table 2; Fig 2C). In level walking, the ankle was the largest contributor to positive mechanical power at 44%, followed by 37% from the hip, and 19% from the knee (rANOVA, *p* = 0.0001; HSD, *p* < 0.0001). As grade increased, the percent contribution of the ankle decreased (rANOVA, *p* < 0.0001) to 34% at 5.71° grade (HSD, *p* = 0.0095) and 28% at 8.53° grade (HSD, *p* < 0.0001) relative to level. Conversely, the percent contribution of the hip increased with grade (rANOVA, *p* < 0.0001) from 37% at level to 47% at 5.71° grade (HSD, *p* = 0.0233) and 52% at 8.53° grade (HSD, *p* < 0.0001). For incline grades, the relative contribution of the knee to positive power was the smallest (19%) and did not change as the power was redistributed primarily between ankle and hip. For decline grades, the only significant shift in percent contribution to positive power was a decrease in the ankle contribution from 44% at level to 34% at -8.53° grade (rANOVA, *p* < 0.0001; HSD, *p* = 0.0167). There was no significant difference in the contribution to positive power among the joints at -8.53° grade.

**Table 1:** Lower-limb joint average mechanical power for walking and running at multiple grades.

	**Grade (deg)**	**Joint Positive Power (W kg**^**-1**^**)**				**Joint Negative Power (W kg**^**-1**^**)**			
		**Ankle**^**##**^	**Knee**^**##**^	**Hip**^**##**^	**Total**^**##**^	**Ankle**^**##**^	**Knee**^**##**^	**Hip**^**##**^	**Total**^**##**^
**Walk** **(1.25 m s**^**-1**^**)**	-8.53	0.30 (0.04)	0.24 (0.03)	0.32 (0.02)	0.86 (0.04)	-0.70 (0.06)	-1.62 (0.14)	-0.28 (0.04)	-2.60 (0.13)
	-5.71	0.41 (0.06)	0.22 (0.03)	0.30 (0.03)	0.94 (0.07)	-0.60 (0.06)	-0.84 (0.14)	-0.16 (0.04)	-1.60 (0.15)
	0	0.45 (0.02)	0.19 (0.01)	0.38 (0.04)	1.02 (0.04)	-0.39 (0.03)	-0.53 (0.06)	-0.11 (0.03)	-1.03 (0.07)
	5.71	0.58 (0.04)	0.32 (0.02)	0.81 (0.07)	1.71 (0.08)	-0.18 (0.01)	-0.45 (0.06)	-0.08 (0.01)	-0.71 (0.07)
	8.53	0.74 (0.04)	0.50 (0.04)	1.36 (0.05)	2.60 (0.08)	-0.15 (0.02)	-0.37 (0.02)	-0.10 (0.01)	-0.62 (0.03)
	**Grade (deg)**	**Joint Positive Power (W kg**^**-1**^**)**				**Joint Negative Power (W kg**^**-1**^**)**			
		**Ankle**^**##**^	**Knee**	**Hip**^**##**^	**Total**^**##**^	**Ankle**	**Knee**^**##**^	**Hip**^**#**^	**Total**^**##**^
**Run** **(2.25 m s**^**-1**^**)**	-5.71	1.28 (0.11)	0.61 (0.07)	0.75 (0.13)	2.64 (0.23)	-1.12 (0.20)	-2.40 (0.1)	-0.37 (0.08)	-3.88 (0.2)
	-2.86	1.54 (0.15)	0.69 (0.13)	0.91 (0.09)	3.14 (0.18)	-0.98 (0.12)	-1.98 (0.20)	-0.29 (0.06)	-3.25 (0.30)
	0	2.01 (0.09)	0.64 (0.08)	1.02 (0.10)	3.66 (0.13)	-1.13 (0.08)	-1.83 (0.10)	-0.15 (0.03)	-3.12 (0.13)
	2.86	2.05 (0.17)	0.66 (0.13)	1.39 (0.20)	4.09 (0.18)	-1.14 (0.10)	-1.57 (0.17)	-0.16 (0.04)	-2.86 (0.20)
	5.71	2.11 (0.23)	0.79 (0.11)	1.63 (0.09)	4.53 (0.19)	-1.07 (0.05)	-1.52 (0.05)	-0.21 (0.03)	-2.81 (0.07)

A repeated measures ANOVA (main effect: grade ^##^p < 0.0001, ^#^p = 0.0281) tested the effect of grade on average joint power. Values reported as mean (s.e.m).

**Table 2:** Percent contribution of each joint to total limb power in walking at 1.25 m s^-1^.

**Joint Positive Power (%)**						
**Grade**	**Ankle**	**Knee**	**Hip**	**Pairwise HSD**		
**(deg)**	^***##***^***p* < 0.0001**	^***##***^***p* = 0.0203**	^***##***^***p* < 0.0001**	***Ank*:*Knee***	***Ank*:*Hip***	***Hip*:*Knee***
-8.53	34 (3)	28 (3)	38 (2)			
	^#^*p* = 0.0167					
-5.71*	43 (4)	25 (3)	32 (3)	*p = 0*.*0031*		
0**	44 (3)	19 (1)	37 (3)	*p < 0*.*0001*		*p = 0*.*0003*
5.71**	34 (2)	19 (1)	47 (2)	*p = 0*.*0001*	*p = 0*.*0009*	*p < 0*.*0001*
	^#^*p* = 0.0095		^#^*p* = 0.0233			
8.53**	29 (2)	19 (1)	52 (1)	*p = 0*.*0001*	*p < 0*.*0001*	*p < 0*.*0001*
	^#^*p* < 0.0001		^#^*p* < 0.0001			
**Joint Negative Power (%)**						
**Grade**	**Ankle**	**Knee**	**Hip**	**Pairwise HSD**		
**(deg)**	^***##***^***p* = 0.0077**	^***##***^***p* = 0.0038**	^***##***^ -	***Ank*:*Knee***	***Ank*:*Hip***	***Hip*:*Knee***
-8.53**	28 (3)	62 (3)	11 (1)	*p < 0*.*0001*	*p = 0*.*0009*	*p < 0*.*0001*
-5.71**	41 (6)	50 (5)	9 (2)		*p = 0*.*0004*	*p < 0*.*0001*
0**	38 (2)	51 (4)	11 (2)	*p = 0*.*0115*	*p < 0*.*0001*	*p < 0*.*0001*
5.71**	27 (2)	62 (2)	11 (1)	*p < 0*.*0001*	*p < 0*.*0001*	*p < 0*.*0001*
		^#^*p* = 0.0433				
8.53**	24 (3)	60 (2)	16 (2)	*p < 0*.*0001*		*p < 0*.*0001*

A repeated measures ANOVA (main effect: grade^##^) tested the effect of grade on stride average joint power distribution of the ankle, knee, and hip (^#^ indicates HSD post-hoc comparison to 0° grade). In addition, a repeated measures ANOVA (main effect: joint*) evaluated the relative contribution of each joint at each grade. (main effect: joint *p = 0.0043; **p < 0.0001). Pairwise HSD was used to evaluate significant differences between joints. Values reported as mean (s.e.m).

#### Negative power

The magnitude of stride average limb negative power decreased with increasing grade (rANOVA, *p* < 0.0001) from -1.03 W kg^-1^ in level to -0.73 W kg^-1^ at 5.71° grade (HSD, *p* = 0.1918) and -0.62 W kg^-1^ at 8.53° grade (HSD, *p* = 0.0305) (Table 1; Fig 2C) Negative limb power was significantly larger in magnitude at -1.60 W kg^-1^ at -5.71° grade (HSD, *p* = 0.0015) and -2.60 W kg^-1^ at -8.53° grade (HSD, *p* < 0.0001). The knee contributed >50% to limb negative power, and the percent contribution was greater than that of the hip in all conditions and that of the ankle in all conditions but the -5.71° grade (rANOVA, p < 0.0001; HSD, *p* < 0.05) (Table 2; Fig 2C). The percent contribution of the knee to negative limb power increased with incline (rANOVA, p = 0.0038) from 51% at level to 63% at 5.71° grade (HSD, *p* = 0.0433) and 60% at 8.53° grade and coincided with a decrease in ankle contribution (rANOVA, p = 0.0007). Ankle negative power contribution was maximized for -5.71° grade at 41%. Hip contribution to negative power did not change with grade and was 12% on average.

### Mechanical power in running

#### Net power

**Fig 3:**
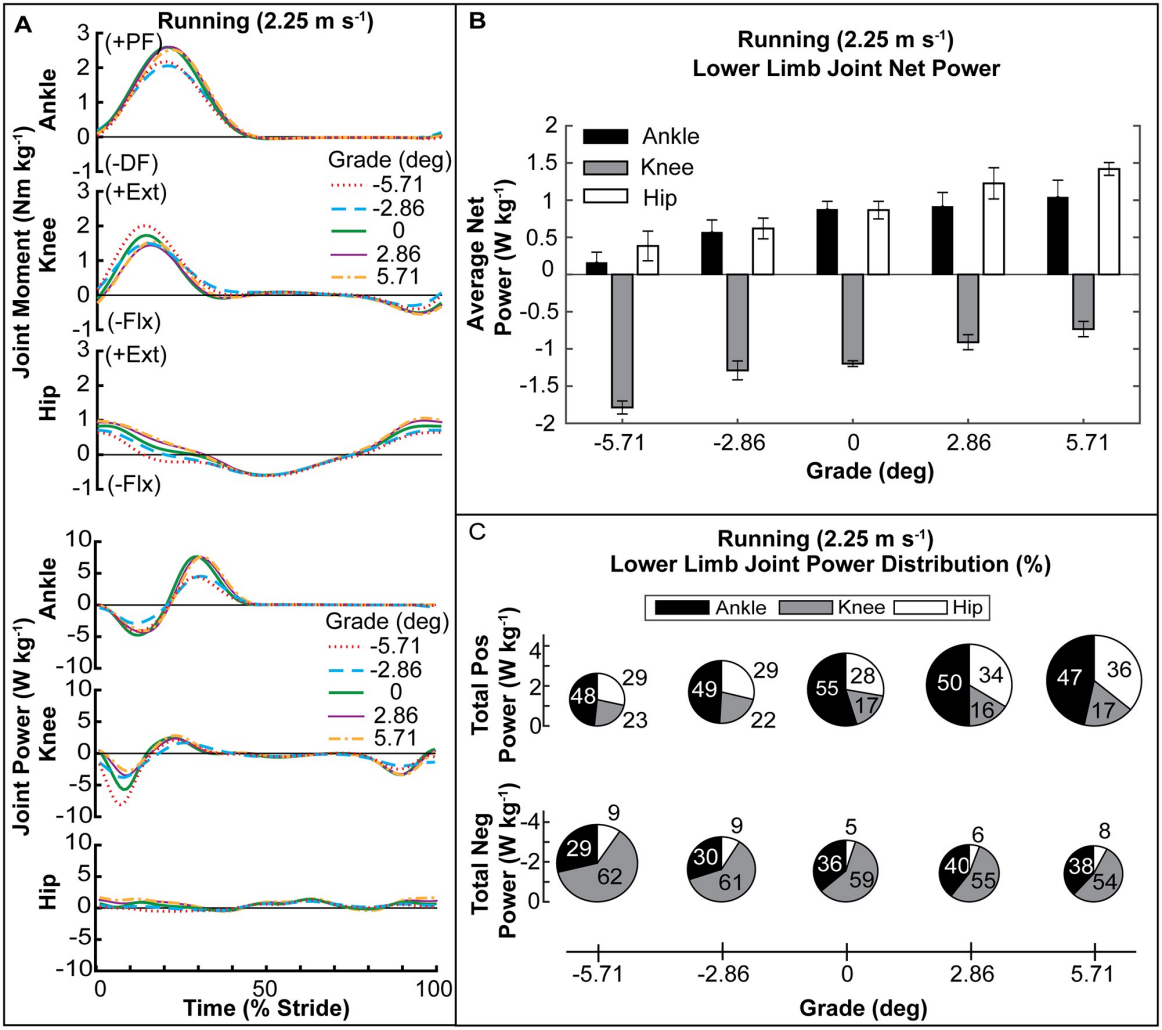
Lower-limb joint kinetics for running at 2.25 m s^-1^ over a range of grades. **(A)** Joint moment and power over a stride. **(B)** Average net power of each joint across grade. **(C)** Percent distribution of average positive and negative lower-limb joint power. The diameter of each pie is normalized to the average positive power at level grade for running (3.66 W kg^-1^).

Joint moments and powers were affected by running grade (Fig 3). Similar to walking, the stride average net power of each joint increased from negative to positive grade (rANOVA, *p* < 0.0001) (Fig 3B). The average net power of the ankle and hip was positive in all conditions and increased in magnitude with increasing grade (rANOVA, *p* < 0.0001). In contrast, the average net power of the knee was negative in all conditions and became more negative in large downhill grades (rANOVA, *p* < 0.0001).

#### Positive power

The average positive power of the limb (ankle + knee + hip) increased with increasing grade (rANOVA, *p* < 0.0001) (Table 1; Fig 3C) from 3.66 W kg^-1^ at level to 4.12 W kg^-1^ and 4.53 W kg^-1^ (HSD, *p* = 0.0005) at 2.86° and 5.71° grades respectively. Limb positive power decreased to 3.14 W kg^-1^ at -2.86°, and to 2.64 W kg^-1^ (HSD, *p* < 0.0001) at -5.71° grade. The ankle was the dominant source of positive mechanical power (>46%) in all conditions and was significantly different from the knee (rANOVA, *p* < 0.0001; HSD, *p* < 0.0001) in all conditions and for the hip in all but the 5.71° grade (rANOVA, *p* < 0.0001; HSD *p* < 0.0171) (Table 3; Fig 3C). With increasing incline, ankle positive power percent contribution decreased (rANOVA, *p* = 0.04) from 55% at level to 46% at 5.71° grade (HSD *p* = 0.0263) while hip contribution increased (rANOVA, *p* = 0.0032) from 28% to 36% in the level versus 5.71° grade condition (HSD, *p* = 0.0051). For decline grades, there was no significant shift in the joint positive power distribution.

**Table 3:** Percent contribution of each joint to total limb power in running at 2.25 m s^-1^.

**Joint Positive Power (%)**						
**Grade**	**Ankle**	**Knee**	**Hip**	**Pairwise HSD**		
**(deg)**	^***##***^***p =* 0.04**	^***##***^***p =* 0.1468**	^***##***^***p =* 0.0032**	***Ank*:*Knee***	***Ank*:*Hip***	***Hip*:*Knee***
-5.71**	48 (3)	23 (2)	28 (4)	*p < 0*.*0001*	*p = 0*.*0002*	
-2.86**	49 (4)	22 (4)	29 (3)	*p <* .*0001*	*p =* .*0023*	
0**	55 (3)	17 (2)	28 (2)	*p <* .*0001*	*p <* .*0001*	*p =* .*0197*
2.86**	50 (4)	16 (3)	33 (4)	*p <* .*0001*	*p =* .*0171*	*p =* .*0186*
5.71**	46 (4)	18 (3)	36 (2)	*p <* .*0001*		*p =* .*0013*
	^#^*p* = 0.0263		^#^*p* = 0.0051			
**Joint Negative Power (%)**						
**Grade**	**Ankle**	**Knee**	**Hip**	**Pairwise HSD**		
**(deg)**	^***##***^***p =* 0.0027**	^***##***^***p =* 0.0094**	^***##***^***p =* 0.1109**	***Ank*:*Knee***	***Ank*:*Hip***	***Hip*:*Knee***
-5.71**	28 (3)	62 (3)	10 (2)	*p <* .*0001*	*p =* .*0003*	*p <* .*0001*
-2.86**	31 (4)	60 (3)	9 (2)	*p <* .*0001*	*p <* .*0001*	*p <* .*0001*
0**	36 (2)	59 (2)	5 (1)	*p <* .*0001*	*p <* .*0001*	*p <* .*0001*
2.86**	41 (5)	54 (4)	5 (1)	*p =* .*0495*	*p <* .*0001*	*p <* .*0001*
5.71**	38 (1)	54 (2)	8 (1)	*p <* .*0001*	*p <* .*0001*	*p <* .*0001*

A repeated measures ANOVA (main effect: grade^##^) tested the effect of grade on stride average joint power distribution of the ankle, knee, and hip (^#^ indicates HSD post-hoc comparison to 0° grade). In addition, a repeated measures ANOVA (main effect: joint*) evaluated the relative contribution of each joint at each grade. (main effect: joint **p < 0.0001). Pairwise HSD was used to evaluate significant differences between joints. Values reported as mean (s.e.m).

#### Negative power

The magnitude of limb negative power in running decreased with grade (rANOVA, *p* < 0.0001) from -3.12 W kg^-1^ at level to -2.86 W kg^-1^ and -2.81 W kg^-1^at 2.86° and 5.71° grade (Table 1; Fig 3C). The limb negative power magnitude increased to -3.25 W kg^-1^ for -2.86° and to -3.88W kg^-1^ for -5.71° grade (HSD, *p* = 0.0002). Similar to walking, each joint contributed different amounts to total limb average negative power (rANOVA *p* < 0.0001) (Table 3; Fig 3C). The knee was the dominant source of negative power, producing >54% for all conditions and contributed significantly more than the ankle or hip (HSD *p* < 0.0001). The ankle contributed approximately 35% of the stride average negative power across all grades and the hip contribution was minimal (~7%).

### Comparisons between walking and running

The average limb positive power was greater in running than walking. Compared to walking, running on level ground resulted in an increase in the ankle’s percent contribution from 44% to 55% (paired t-test *p* = 0.0024) and a decrease in the hip’s percent contribution from 37% to 28% (paired t-test *p* = 0.0196). The trend was similar at 5.71° grade, where compared to walking, running resulted in an increase in the ankle’s percent contribution from 34% to 46% (paired t-test *p* = 0.0024) and a decrease in the hip’s percent contribution from 47% to 36% (paired t-test *p* = 0.0196). While the ankle was the dominant contributor to positive power in walking at 5.71° grade, the hip was the dominant contributor to positive power for running at 5.71° grade. For negative power at the 5.71° grade, compared to walking, running resulted in an increase in the ankle’s percent negative contribution from 27% to 38% (paired t-test *p* = 0.001) and a decrease in the knee’s percent contribution from 62% to 54% (paired t-test *p* = 0.0338).

Additional text and data regarding participants metabolic energy consumption are provided in S1 File. Similar to Margaria *et al*. [34], we found the greatest efficiency of positive work at -5.71° slope for both walking and running. Additionally, the efficiency of positive work during walking at the extreme uphill (+8.53°) was ~0.25 reflecting the efficiency of muscle-tendons during tasks exhibiting predominantly positive work [33, 46–49].

## Discussion

Our aim in this study was to measure and analyze human biomechanical response during walking and running on sloped surfaces in order to build a roadmap to help guide development of lower-limb wearable exoskeletons capable of adjusting to changing mechanical demands in real-world environments. We characterized the distribution of positive and negative mechanical power output across the lower-limb joints for incline and decline grades during walking and running. Our results confirm and are supported by previous studies demonstrating that the required mechanical power from the lower limbs is heavily dependent on both ground slope and gait [35–45, 50–53]. Energy must be injected or extracted to raise or lower the potential energy of the center of mass (COM) for incline/decline walking [33, 34]. Our data confirm that in both walking and running gait, the stride average total limb (ankle + knee + hip) power changes from net negative on decline grades to net positive on incline grades (Figs 2B and 3B, Table 1). In addition, our results support the hypotheses that limb-joint positive mechanical power would shift to the hip for incline walking (Fig 2C, Tables 1 and 2) and running (Fig 3C, Tables 1 and 3). However, the ankle remained the dominant source of positive mechanical power in incline running for the tested grades (Fig 3C, Table 3). For decline walking and running, our results did not support the hypothesis that the distribution of limb-joint negative mechanical power would shift even further to the knee. While there was an increase in the magnitude of negative mechanical power produced at the knee, and the knee was the dominant source of limb-joint negative mechanical power, there was not a significant increase in the percent contribution in decline grades (Figs 2, 3, Tables 2 and 3).

### Limb-joint contributions in walking and running

#### Walking positive power

The 44% contribution of the ankle towards positive power that we measured for level walking at 1.25 m s^-1^ (Fig 2, Table 2) was greater than the 40% found by Farris and Sawicki [54] but less than the 51% and 55% measured by Montgomery and Grabowski [42] and Alexander et al [55]. Our methodology was more similar to Farris and Sawicki [54]. A point to consider is that Montgomery and Grabowski [42] and Alexander et al [55] analyzed joint work while we analyzed average joint power. This may affect direct comparisons of trends in joint work (J kg^-1^) and joint power (J s^-1^ kg^-1^) across grades as stride time can change. However, for comparing percent contribution, they should be identical as the effect of stride time is negated in dividing the joint power/work by limb power/work. The walking grades in our study (±5.71° (10%) and ±8.53° (15%)) were similar to the steeper grades used by Montgomery and Grabowski [42] (±3° and ±6°) and the 6° grade in Alexander et al [55]. Alexander et al [55] studied a slightly slower overground speed of 1.1 m s^-1^.

Our findings agree with previous studies demonstrating that for incline walking the hip becomes an important source of positive mechanical power generation [24, 36, 38, 39, 42]. We found that the ankle contribution decreased with increasing grade, and similar to Montgomery and Grabowski [42], the hip contribution increased with grade and was the dominant source of positive power at all the incline grades (Fig 2C, Tables 1 and 2).

#### Walking negative power

Our data are is agreement with previous studies that demonstrated that in decline walking the knee is the primary source of negative mechanical power generation [39, 55] and that the contribution increases with larger declines [55] (Fig 2C, Tables 1 and 2). These findings are however in contrast with Montgomery and Grabowski [42] which showed that the ankle contributed more to negative power at level and up to -6° grade and no effect of decline on knee negative power.

#### Running positive power

The 55% contribution of the ankle towards positive power that we measured for level running at 2.25 m s^-1^ (Fig 3, Table 3) was greater than the 45% found by Farris and Sawicki [54]. We again found evidence of a redistribution of positive work to the hip and away from the ankle during uphill running. However, in running, the ankle still produced 46% of the positive power at 5.71° uphill grade and there was not a significant difference between ankle and hip (Fig 3C, Table 3). This finding seems to be in slight contrast to previous study which showed that the hip contributed most to the increase in work for incline running [35]. However, our results may differ due to the different grade (6° and 12°), faster speed (3.0 and 3.5 m s^-1^), and lack of treadmill use in [35].

#### Running negative power

Our data are is agreement with previous studies that demonstrated that the knee is the primary source of negative mechanical power generation (Fig 3C, Table 3) [40].

### Relationship between structure and function across task demand

The functional role of the ankle and the hip across grades aligns with the physiological structure of each joint’s muscle-tendon units (MTs). The hip MTs have short tendons and long muscle fascicles with low pennation [56]. In contrast, the structure of the ankle plantarflexor MTs, comprises relatively short, pennate muscle fibers in series with long compliant tendons. Added compliance in distal MTs make them ideal for storage and return of elastic energy during the gait cycle [56–58]. In incline gait, mechanical energy must be added to the body. Prior studies suggest that the structure of the MTs in the more proximal joints (*i*.*e*., hip) may be better suited to performing work on the COM because short, stiff tendons can directly transmit the work of the muscles to the joint [56]. Furthermore, long muscle fascicles allow for production of force over a relatively larger range of motion and are important in incline walking due to larger joint range of motion [42].

In line with the idea that structure drives function, our walking data demonstrate a shift to power output in more proximal joints with an increase in incline (Fig 2C). This finding is similar to prior studies which also show the dominant source of positive mechanical power shifts from the ankle to the hip in uphill walking [24, 36, 42]. Unlike walking, in running the ankle continues to be the dominant producer of positive power up to 5.71° and there is almost no change in negative joint power at the ankle (Fig 3C). This trend suggests that energy cycling through elastic mechanisms may still be an important feature retained in uphill running [59]. Due to the need for faster acceleration of the body in uphill running, ankle joint elasticity may facilitate higher peak powers and more net work output from the plantarflexors [56] by decreasing the required shortening velocity of the muscle fascicles of the ankle. Indeed, *in vivo* studies where ultrasound images of the triceps surae were taken in running and walking showed series elastic tissues allow the muscles to operate at lower average shortening velocities and that elastic recoil contributes substantially to positive work [32]. Additional *in vivo* studies of human muscle function, especially at proximal joints, in uphill and downhill walking and running would shed light on how MT architecture interacts with task demand for mechanical power generation /dissipation.

### Balance of positive/negative power varies across limb-joints and grades

Net mechanical power production of the limb was governed by a balance between positive and negative power output that varied from joint to joint (Figs 2B and 3B). The hip’s contribution to walking and running on sloped surfaces was net positive across all grades and gaits we tested and was modulated predominantly by changes in production of positive power (Figs 2 and 3, Tables 1–3). Despite producing net positive power output across grades, the hip was not the largest absolute contributor of positive power in most conditions (except incline walking). This was because the hip contributes mostly positive power and very little negative power across conditions. In comparison, the ankle produces large amounts of both positive and negative power which offset and results in lower net power.

Conversely, the change in the knee net power seen across grades was affected heavily by changes in knee negative power and less so by changes in positive power. The knee was the dominant contributor (>50%) to negative power across all grades in both walking and running (Figs 2C and 3C, Tables 1–3). In all except the highest incline walking grade, the knee produced more negative than positive power, resulting in negative net power (Figs 2B and 3B, Table 1).

At the ankle, lower-limb joint power production across grade/gait was more evenly distributed between positive and negative power in comparison to the hip (positive work modulated) and knee (negative work modulated). The average net power of the ankle was generated by adjustments to both positive and negative power across grade and gait. (Figs 2 and 3 Table 1) In level walking, the net power from the ankle was smaller than the hip despite the larger contribution to positive power from the ankle (Fig 2, Tables 1 and 2). During incline walking, the ankle’s percent contribution to both positive and negative power decreased, potentially reflecting a reduced capacity for energy recycling. In decline walking, we observed the opposite trend where ankle net power was negative reflecting an increased capacity to store energy. In running, the ankle was the dominant source of positive mechanical power across all grades and the net power of the ankle was positive for all grades. (Fig 3, Tables 1 and 3).

### Implications for lower-limb exoskeleton development

**Fig 4:**
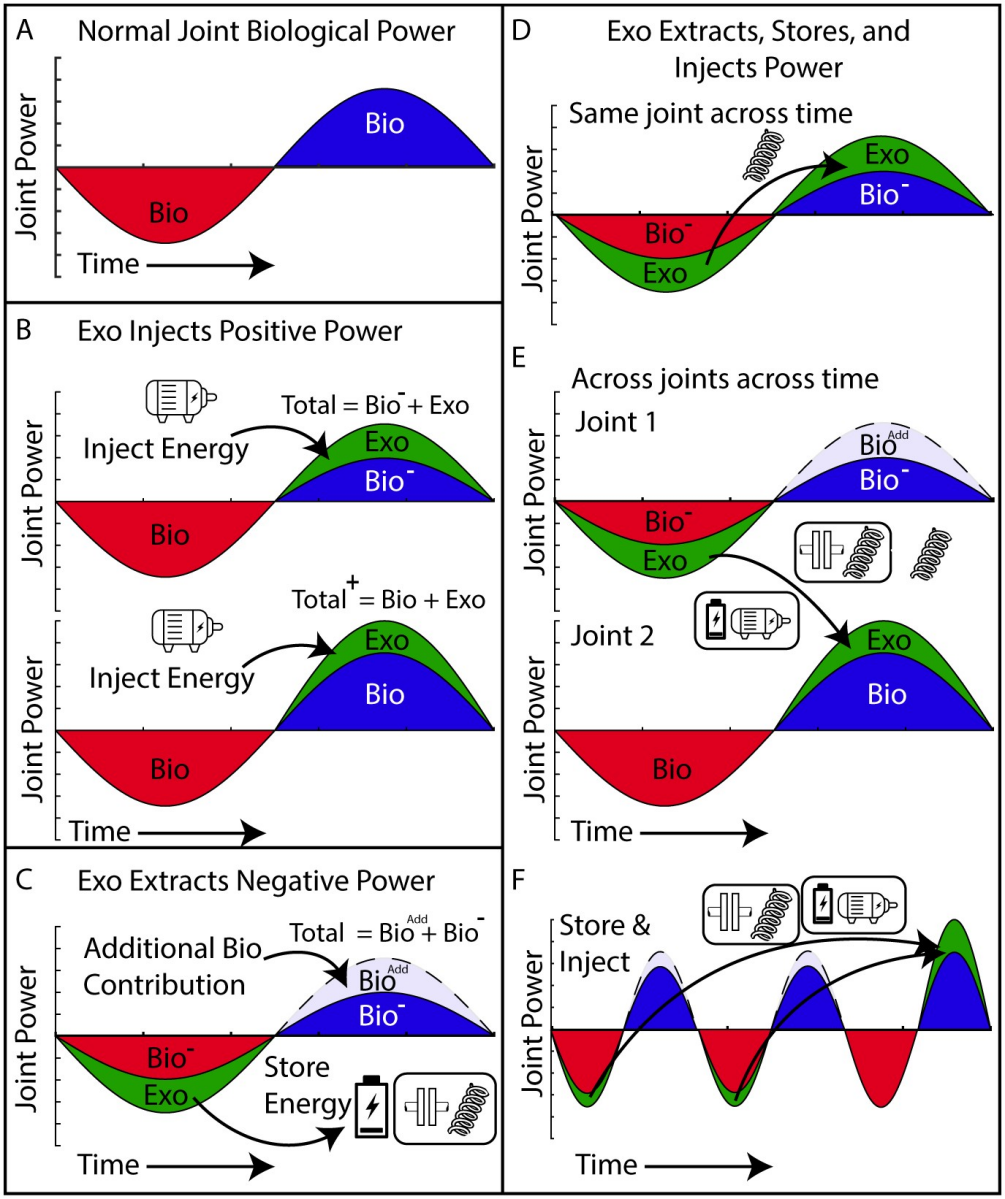
Potential mechanisms for exoskeleton energy exchange. **(A)** Example of energy cycle for a joint where negative joint power (red) is followed by positive joint power (blue) similar to the ankle power during gait. **(B)** The exoskeleton (exo) (green) produces positive power and injects energy at the joint during the positive power phase of the gait via a motor or some other energy source. (Top) The positive bio power (bio) is reduced such that the total (bio+exo) positive power output of the joint remains the same (*i*.*e*., replacement). (Bottom) The additional energy increases the total (bio+exo) positive power output of the joint (*i*.*e*., augmentation). This is the most common mode employed on powered exoskeletons [4, 7, 8, 15, 18]. **(C)** The exoskeleton (green) produces negative power and extracts energy from the joint during the negative power phase of the gait via a damper or some other energy sink and, in this example, the user maintains the total (exo+ bio) negative power output of the joint, enabling a reduced biological contribution (*i*.*e*., replacement). In this mode, the exoskeleton negative power could drive an electrical generator and energy could be stored in a battery or used to power electronic devices [19, 60, 61]. If the negative power is normally recycled within the body and transferred to the positive power phase, additional biological power may be required to maintain biological positive power output (Bio^Add^). **(D-F)** The exoskeleton (green) could also operate in transfer mode by sequencing extraction and injection phases within or across the joints over time. **(D)** In the simplest case the exoskeleton stores energy during the negative power phase and returns it immediately to the same joint (*e*.*g*., with a spring) and, in this example, the user maintains the total joint power output enabling a reduction in both biological positive and negative power (= replacement) [26]. Other variants on transfer mode include: **(E)** The exoskeleton extracts energy at one joint (similar to C) and then immediately injects it at another (similar to B) [3]. **(F)** The exoskeleton extracts energy at one joint (*e*.*g*., with a spring or generator), temporarily stores it (*e*.*g*., using a battery or a clutch) and then after some delay injects it at the same joint (*e*.*g*., using a motor powered by the battery or spring recoil on release of a clutch).

How the biological system distributes power across the joints in a variety of gait conditions has important implications for development of wearable assistive devices. To develop a roadmap for lower-limb exoskeleton design, we first define three main modes of operation: 1) (Net +) Energy injection–the device adds mechanical energy to the gait cycle using external sources of energy; 2) (Net -) Energy extraction–the device removes mechanical energy from the gait cycle to be dissipated as heat or stored (*e*.*g*., as mechanical energy in a spring or electrical energy in a battery); 3) (Net 0) Energy transfer–the device extracts energy at one time during gait and then injects it within or across joints at some time later (Fig 4). With these modes the energy which is added, removed, or transferred may have different effects on the user’s biological and total joint power outputs, and, while most studies have a goal in mind (*e*.*g*., reduce biological moments and powers), the effects are often non-intuitive and hard to predict. Because the effect of an assistive device on the user is heavily dependent on the individual user’s biomechanical response, there are three potentially likely biomechanical outcomes resulting from any of these modes of operation. The magnitude of the user’s biological joint power could: O1) decrease (= replacement) O2) remain constant (= augmentation), or O3) increase (= enhancement). We focus on the possible physiological response outcomes (O1-3) for devices that inject positive power in this work, but the same principles also apply for the other device modes as well (*i*.*e*., extraction and transfer).

#### Energy injection

The first mode of device operation entails adding positive mechanical work at a joint(s) when the joint is producing positive power. This is the most prevalent strategy used in exoskeletons targeting the hip, knee, and ankle with the common desired goal being the reduction of metabolic demand in healthy individuals [4, 7, 8, 15, 18, 25, 62, 63]. The common expectation is the outcome where the addition of mechanical power causes a concomitant reduction of biological power while total power mostly remains constant (O1: replacement). While it’s been demonstrated that users will reduce biological moment such that the total joint moment remains invariant [64, 65], reductions in biological power often do not reflect full replacement [18, 66]. Thus, unlike what might be desired, the second physiological response outcome is often observed. Here, the biological power is reduced by less than the exoskeleton injects and the magnitude of the total joint power is increased (O2: augmentation) (Fig 4B) [8]. [18, 66]. The third physiological response outcome is that the addition of exoskeleton positive power causes an enhancement of the biological power (O3: enhancement). It is possible that when injecting positive exoskeleton power, the user actually increases their biological power output and thus enhances the total joint power beyond the exoskeleton’s contribution. So far, we are not aware of cases where this physiological response has occurred, but it would be desirable for assistive and rehabilitative technology intended to improve function in clinical populations with baseline deficits in limb and joint power output (*e*.*g*., post-stroke) [67]. For example, the addition of positive power during push-off may help promote the recruitment of weak plantarflexors in stroke survivors or older adults. Studies have begun to demonstrate the potential for enhancing performance in clinical populations by providing positive power to the ankle [30, 68], however the actual effect on biological power is still unclear.

The insights from this study into the biomechanical strategies used by individuals in changing gait may be used to guide strategies for assistive devices that inject positive power. The most notable example comes from the observed shift to hip dominated positive power in walking uphill (Fig 2). Given limited power supply of the device, our data would suggest that assistance should be redirected away from the ankle to the hip when transitioning to incline walking. Conversely, for running (Fig 3), the ankle is the largest contributor to positive average power across *all* slopes and thus, shifting assistance to the hip may not be as beneficial.

#### Energy extraction

The second mode of device operation involves removing negative mechanical work at a joint(s) when the joint is producing negative power. The extracted mechanical energy could be dissipated as heat (*e*.*g*., in a damper) or harvested to generate electricity which can then be stored in a battery or used to power electronic devices (Fig 4C). Additionally, an exoskeleton that effectively extracts energy from the gait cycle can potentially reduce the negative power required from muscles which, unlike many mechanical systems, require energy to elongate under load [69]. Similar to the effects from injecting positive power, generating negative power with exoskeletons may have a range of effects on the biological system that can be non-intuitive. For example, if an exoskeleton offloads a portion of the negative biological power at a joint, and that power was derived from stored energy in elastic tissues which can no longer be returned, it is possible that additional biological power may need to be generated in the positive phase to make up for lost energy stores (Fig 4C). However, in the nominal case where the negative biological power is merely dissipated as heat rather than recycled, then the reduction in total power during the latter half of the cycle may not be problematic as the loss of energy doesn’t need to be compensated for.

The knee has been the focus of energy harvesting exoskeletons due to its production of substantial negative power in gait, especially near the end of swing phase of walking (Fig 2). There are several indications that if done correctly it is possible to generate electrical energy while reducing the muscle energetic demands and whole body metabolic cost [19, 60, 70, 71]. With consideration to changing mechanical demands on sloped surfaces, our results suggest potential for harvesting energy using a knee exoskeleton during decline walking due to large increases in knee negative power throughout the gait cycle (Fig 2). In running, a knee exoskeleton may be widely versatile because the knee generates a large amount of negative power across all slopes including on inclines (Fig 3).

Although the ankle produces substantial negative power, harvesting exoskeletons might be ineffective in level gait because much of the joint power is recycled in elastic tissues [32], and thus as mentioned previously, the biological system would need to replace these losses with muscle work during a positive power phase at some joint in the limb. However, because ankle negative power increases and positive power decreases on declined surfaces (Fig 2), energy harvesting may be a viable candidate at the ankle for decline walking.

#### Energy transfer

The third mode of device operation is to transfer energy from one phase to another across the gait cycle either within or across joints (Fig 4D–4F). In this mode, because the exoskeleton extracts energy in the negative phase (*e*.*g*., Fig 4C) and then injects the same energy later (*e*.*g*., Fig 4B) in a positive phase , external power consumption of the device can be minimized (*e*.*g*., by using passive elements like springs and clutches) [72]. In addition, intra-joint transfer of energy from a negative power phase to a positive power phase may help mitigate the complication of the reduced biological energy storage because the mechanical power is returned in the latter half of the power cycle. As depicted in Fig 4D, it is possible that the total power output of the joint (exo+bio) remains constant despite the reduction of biological power in both the negative and positive power phases. The simplest device applying this mode of operation is an elastic exoskeleton that uses a spring in parallel with the biological plantarflexors to store energy (negative biological power) which is returned later in stance (positive biological power) as done by Collins, Wiggin, and Sawicki [26]. According to our data, while this approach of storing and returning energy at the ankle can be effective for level ground gaits, at other grades the strategy of immediate storage and return of mechanical energy may not be as effective. Adding a spring in parallel on inclines or declines would likely require an additional biological energy source to inject/extract energy elsewhere in the stride. Another option is to transfer power across joints as depicted in Fig 4E (*i*.*e*., inter-joint transfer). One example is the storage of energy from knee deceleration in late swing and releasing it at the ankle during push-off [3]. From our data, we additionally show that energy storage in the knee during early stance and releasing it at the ankle during push-off becomes increasingly viable with decreasing grade (Figs 2 and 3). A final scenario is that the power from the negative phase could be temporarily stored via battery or clutch and returned at a later time–an approach that has been used within a single gait cycle in foot-ankle prosthesis designs [73, 74]. This last approach, extraction, storage, and then delayed release (Fig 4F) opens up the possibility to store energy over multiple cycles, perhaps accumulating it, and then returning it in a single large burst over a shorter time period to achieve power amplification that may be necessary for on-off accelerations or maximum effort jumps [75].

#### Limitations

Our sample size (N = 8) was lower than comparable studies, (e.g., N = 20 in Montgomery and Grabowski [42]). We were only able to compare walking and running mechanics at 0 and ±5.71°. The running speed we studied, 2.25 m s^-1^, is slow and may complicate generalizing our findings to faster running speeds. However, for running on the level, a previous study has shown that the joint power distribution does not change from 2.25 to 3.25 m s^-1^ [54]. Thus, for running, joint power distribution may not vary much and magnitudes across limb-joints may scale with total limb power. In this work we focus our discussion on how exoskeleton power injection, extraction, and transfer may influence a limb- joints locally, but it is important to note that there may be a cascade of complicated inter-joint interactions along the kinematic chain that could lead to unexpected limb-level outcomes that will require advanced analysis to disentangle. Finally, we note that here we have developed a framework for exoskeletons which operate in parallel with biological muscles and tendons. The guide for development of prostheses, which operate in series with biological structures and aim to emulate or fully replace biological joint function, may differ [76].

## Conclusions

Locomotion in the ‘real-world’ involves adjusting speed, changing gait from walk to run and moving up or downhill. The purpose of this study was to characterize changes in lower-limb joint kinetics for walking and running over a range of ground slopes. Specifically, we sought to understand how each joint contributed to total limb positive, negative, and net power output in order to guide development of exoskeleton actuation schemes capable of handling ‘real-world’ mechanical demands. Results of limb-joint level energy analyses motivated us to define three operating modes that exoskeletons could employ: 1) Energy injection: Addition of positive power during positive joint power phases, 2) Energy extraction: Removal of negative power (*i*.*e*., energy harvesting) during negative joint power phase. 3) Energy transfer: extracting energy from one phase and injecting it in another phase at some time later. An important next step will be to examine whether using biological patterns of joint power output as a ‘road-map’ to apply the three exoskeleton operating modes can improve walking and running performance (*e*.*g*., reduced metabolic cost) on fixed or time varying uphill and downhill slopes.

## Supporting information

S1 File (DOCX)

## References

[1] Beck ON, Punith LK, Nuckols RW, Sawicki GS. Exoskeletons Improve Locomotion Economy by Reducing Active Muscle Volume. Exercise and sport sciences reviews. 2019;47(4):237–45. 10.1249/JES.0000000000000204 31436749

[2] Sawicki GS, Beck ON, Kang I, Young AJ. The exoskeleton expansion: improving walking and running economy. J Neuroeng Rehabil. 2020;17(1):25 Epub 2020/02/23. 10.1186/s12984-020-00663-9 . 32075669 PMC7029455

[3] Malcolm P, Derave W, Galle S, De Clercq D. A simple exoskeleton that assists plantarflexion can reduce the metabolic cost of human walking. PloS one. 2013;8(2):e56137 10.1371/journal.pone.0056137 23418524 PMC3571952

[4] Mooney LM, Rouse EJ, Herr HM. Autonomous exoskeleton reduces metabolic cost of human walking. J Neuroeng Rehabil. 2014;11:151 Epub 2014/11/05. 10.1186/1743-0003-11-151 . 25367552 PMC4236484

[5] Galle S, Malcolm P, Derave W, De Clercq D. Enhancing performance during inclined loaded walking with a powered ankle-foot exoskeleton. Eur J Appl Physiol. 2014;114(11):2341–51. 10.1007/s00421-014-2955-1 . 25064193

[6] Collins SH, Wiggin MB, Sawicki GS. Reducing the energy cost of human walking using an unpowered exoskeleton. Nature. 2015;522(7555):212–5. 10.1038/nature14288 . 25830889 PMC4481882

[7] Zhang J, Fiers P, Witte KA, Jackson RW, Poggensee KL, Atkeson CG, et al Human-in-the-loop optimization of exoskeleton assistance during walking. Science. 2017;356(6344):1280–4. 10.1126/science.aal5054 . 28642437

[8] Koller JR, Jacobs DA, Ferris DP, Remy CD. Learning to walk with an adaptive gain proportional myoelectric controller for a robotic ankle exoskeleton. J Neuroeng Rehabil. 2015;12(1):97 10.1186/s12984-015-0086-5 . 26536868 PMC4634144

[9] Nuckols RW, Dick TJM, Beck ON, Sawicki GS. Ultrasound imaging links soleus muscle neuromechanics and energetics during human walking with elastic ankle exoskeletons. Scientific reports. 2020;10(1):3604 10.1038/s41598-020-60360-4 32109239 PMC7046782

[10] Nuckols RW, Sawicki GS. Impact of elastic ankle exoskeleton stiffness on neuromechanics and energetics of human walking across multiple speeds. J Neuroeng Rehabil. 2020;17(1):75 Epub 2020/06/17. 10.1186/s12984-020-00703-4 . 32539840 PMC7294672

[11] Lee S, Kim J, Baker L, Long A, Karavas N, Menard N, et al Autonomous multi-joint soft exosuit with augmentation-power-based control parameter tuning reduces energy cost of loaded walking. Journal of neuroengineering and rehabilitation. 2018;15(1):66 10.1186/s12984-018-0410-y 30001726 PMC6044002

[12] Witte KA, Fiers P, Sheets-Singer AL, Collins SH. Improving the energy economy of human running with powered and unpowered ankle exoskeleton assistance. Science Robotics. 2020;5(40):eaay9108. 10.1126/scirobotics.aay9108 33022600

[13] Galle S, Malcolm P, Derave W, De Clercq D. Adaptation to walking with an exoskeleton that assists ankle extension. Gait & posture. 2013;38(3):495–9. 10.1016/j.gaitpost.2013.01.029 23465319

[14] Zhang J, Cheah CC, Collins SH, editors. Experimental comparison of torque control methods on an ankle exoskeleton during human walking. Robotics and Automation (ICRA), 2015 IEEE International Conference on; 2015: IEEE.

[15] Sawicki GS, Ferris DP. Mechanics and energetics of level walking with powered ankle exoskeletons. J Exp Biol. 2008;211(Pt 9):1402–13. 10.1242/jeb.009241 . 18424674

[16] Galle S, Derave W, Bossuyt F, Calders P, Malcolm P, De Clercq D. Exoskeleton plantarflexion assistance for elderly. Gait Posture. 2017;52:183–8. 10.1016/j.gaitpost.2016.11.040 . 27915222

[17] Galle S, Malcolm P, Collins SH, De Clercq D. Reducing the metabolic cost of walking with an ankle exoskeleton: interaction between actuation timing and power. J Neuroeng Rehabil. 2017;14(1):35 Epub 2017/04/30. 10.1186/s12984-017-0235-0 . 28449684 PMC5408443

[18] Quinlivan B, Lee S, Malcolm P, Rossi D, Grimmer M, Siviy C, et al Assistance magnitude versus metabolic cost reductions for a tethered multiarticular soft exosuit. Science Robotics. 2017;2(2):eaah4416. 10.1126/scirobotics.aah4416 33157865

[19] Donelan JM, Li Q, Naing V, Hoffer JA, Weber DJ, Kuo AD. Biomechanical energy harvesting: generating electricity during walking with minimal user effort. Science. 2008;319(5864):807–10. 10.1126/science.1149860 . 18258914

[20] Ding Y, Galiana I, Asbeck AT, Quinlivan B, De Rossi SMM, Walsh C, editors. Multi-joint actuation platform for lower extremity soft exosuits. Robotics and automation (ICRA), 2014 IEEE international conference on; 2014: IEEE.

[21] Ding Y, Panizzolo FA, Siviy C, Malcolm P, Galiana I, Holt KG, et al Effect of timing of hip extension assistance during loaded walking with a soft exosuit. J Neuroeng Rehabil. 2016;13(1):87 Epub 2016/10/08. 10.1186/s12984-016-0196-8 . 27716439 PMC5048481

[22] Ding Y, Kim M, Kuindersma S, Walsh CJ. Human-in-the-loop optimization of hip assistance with a soft exosuit during walking. Science Robotics. 2018;3(15):eaar5438. 10.1126/scirobotics.aar5438 33141683

[23] Nuckols RW S G. Impact of elastic ankle exoskeleton stiffness on neuromechanics and energetics of human walking across multiple speeds. In Review at Journal of Neuroengineering and Rehabilitation. 2020 10.21203/rs.2.20510/v1. PMC7294672 32539840

[24] Sawicki GS, Ferris DP. Mechanics and energetics of incline walking with robotic ankle exoskeletons. J Exp Biol. 2009;212(Pt 1):32–41. 10.1242/jeb.017277 . 19088208

[25] Mooney LM, Rouse EJ, Herr HM. Autonomous exoskeleton reduces metabolic cost of human walking during load carriage. J Neuroeng Rehabil. 2014;11:80 10.1186/1743-0003-11-80 . 24885527 PMC4036406

[26] Collins SH, Wiggin MB, Sawicki GS. Reducing the energy cost of human walking using an unpowered exoskeleton. Nature. 2015 10.1038/nature14288 25830889 PMC4481882

[27] Nuckols RW, Dick TJM, Beck ON, Sawicki GS. Ultrasound imaging links soleus muscle neuromechanics and energetics during human walking with elastic ankle exoskeletons. Scientific reports. 2020;10(1):3604 Epub 2020/02/29. 10.1038/s41598-020-60360-4 . 32109239 PMC7046782

[28] Kuo AD, Donelan JM, Ruina A. Energetic consequences of walking like an inverted pendulum: step-to-step transitions. Exerc Sport Sci Rev. 2005;33(2):88–97. 10.1097/00003677-200504000-00006 . 15821430

[29] Takahashi KZ, Lewek MD, Sawicki GS. A neuromechanics-based powered ankle exoskeleton to assist walking post-stroke: a feasibility study. J Neuroeng Rehabil. 2015;12(1):23 10.1186/s12984-015-0015-7 . 25889283 PMC4367918

[30] Awad LN, Bae J, O'Donnell K, De Rossi SMM, Hendron K, Sloot LH, et al A soft robotic exosuit improves walking in patients after stroke. Science translational medicine. 2017;9(400). 10.1126/scitranslmed.aai9084 . 28747517

[31] Ishikawa M, Komi PV, Grey MJ, Lepola V, Bruggemann GP. Muscle-tendon interaction and elastic energy usage in human walking. J Appl Physiol (1985). 2005;99(2):603–8. 10.1152/japplphysiol.00189.2005 . 15845776

[32] Farris DJ, Sawicki GS. Human medial gastrocnemius force-velocity behavior shifts with locomotion speed and gait. Proc Natl Acad Sci U S A. 2012;109(3):977–82. 10.1073/pnas.1107972109 . 22219360 PMC3271879

[33] Minetti AE, Moia C, Roi GS, Susta D, Ferretti G. Energy cost of walking and running at extreme uphill and downhill slopes. Journal of applied physiology (Bethesda, Md: 1985). 2002;93(3):1039–46. 10.1152/japplphysiol.01177.2001 12183501

[34] Margaria R, Cerretelli P, Aghemo P, Sassi G. Energy cost of running. J Appl Physiol. 1963;18:367–70. 10.1152/jappl.1963.18.2.367 . 13932993

[35] Roberts TJ, Belliveau RA. Sources of mechanical power for uphill running in humans. J Exp Biol. 2005;208(Pt 10):1963–70. 10.1242/jeb.01555 . 15879076

[36] Lay AN, Hass CJ, Gregor RJ. The effects of sloped surfaces on locomotion: a kinematic and kinetic analysis. J Biomech. 2006;39(9):1621–8. 10.1016/j.jbiomech.2005.05.005 . 15990102

[37] Silder A, Besier T, Delp SL. Predicting the metabolic cost of incline walking from muscle activity and walking mechanics. J Biomech. 2012;45(10):1842–9. 10.1016/j.jbiomech.2012.03.032 . 22578744 PMC4504736

[38] Franz JR, Kram R. Advanced age and the mechanics of uphill walking: a joint-level, inverse dynamic analysis. Gait Posture. 2014;39(1):135–40. 10.1016/j.gaitpost.2013.06.012 . 23850328 PMC3842369

[39] DeVita P, Helseth J, Hortobagyi T. Muscles do more positive than negative work in human locomotion. The Journal of experimental biology. 2007;210(Pt 19):3361–73. 10.1242/jeb.003970 17872990 PMC2577758

[40] Devita P, Janshen L, Rider P, Solnik S, Hortobagyi T. Muscle work is biased toward energy generation over dissipation in non-level running. Journal of Biomechanics. 2008;41(16):3354–9. 10.1016/j.jbiomech.2008.09.024 19010471 PMC2590776

[41] Alexander N, Strutzenberger G, Ameshofer LM, Schwameder H. Lower limb joint work and joint work contribution during downhill and uphill walking at different inclinations. Journal of biomechanics. 2017;61:75–80. 10.1016/j.jbiomech.2017.07.001 28734544

[42] Montgomery JR, Grabowski AM. The contributions of ankle, knee and hip joint work to individual leg work change during uphill and downhill walking over a range of speeds. Royal Society open science. 2018;5(8):180550 10.1098/rsos.180550 30225047 PMC6124028

[43] Pickle NT, Grabowski AM, Auyang AG, Silverman AK. The functional roles of muscles during sloped walking. Journal of biomechanics. 2016;49(14):3244–51. 10.1016/j.jbiomech.2016.08.004 27553849 PMC5167499

[44] Jeffers JR, Auyang AG, Grabowski AM. The correlation between metabolic and individual leg mechanical power during walking at different slopes and velocities. Journal of biomechanics. 2015;48(11):2919–24. 10.1016/j.jbiomech.2015.04.023 25959113 PMC4893810

[45] Vernillo G, Giandolini M, Edwards WB, Morin J-B, Samozino P, Horvais N, et al Biomechanics and physiology of uphill and downhill running. Sports Medicine. 2017;47(4):615–29. 10.1007/s40279-016-0605-y 27501719

[46] Margaria R. Positive and negative work performances and their efficiencies in human locomotion. Internationale Zeitschrift fur angewandte Physiologie, einschliesslich Arbeitsphysiologie. 1968;25(4):339–51. 10.1007/BF00699624 . 5658204

[47] Davies CT, Barnes C. Negative (eccentric) work. II. Physiological responses to walking uphill and downhill on a motor-driven treadmill. Ergonomics. 1972;15(2):121–31. 10.1080/00140137208924416 . 5036082

[48] Minetti AE, Ardigo LP, Saibene F. Mechanical determinants of gradient walking energetics in man. J Physiol. 1993;472:725–35. 10.1113/jphysiol.1993.sp019969 . 8145168 PMC1160509

[49] Zai CZ, Grabowski AM. The metabolic power required to support body weight and accelerate body mass changes during walking on uphill and downhill slopes. Journal of Biomechanics. 2020:109667. 10.1016/j.jbiomech.2020.109667 32063278

[50] Lee DV, McGuigan MP, Yoo EH, Biewener AA. Compliance, actuation, and work characteristics of the goat foreleg and hindleg during level, uphill, and downhill running. Journal of applied physiology. 2008;104(1):130–41. 10.1152/japplphysiol.01090.2006 17947498 PMC2413412

[51] McGuigan MP, Yoo E, Lee DV, Biewener AA. Dynamics of goat distal hind limb muscle–tendon function in response to locomotor grade. Journal of Experimental Biology. 2009;212(13):2092–104. 19525436 10.1242/jeb.028076 PMC2702455

[52] Qiao M, Abbas JJ, Jindrich DL. A model for differential leg joint function during human running. Bioinspiration & biomimetics. 2017;12(1):016015. 28134133 10.1088/1748-3190/aa50b0

[53] Qiao M, Jindrich DL. Leg joint function during walking acceleration and deceleration. Journal of biomechanics. 2016;49(1):66–72. 10.1016/j.jbiomech.2015.11.022 26686397

[54] Farris DJ, Sawicki GS. The mechanics and energetics of human walking and running: a joint level perspective. J R Soc Interface. 2012;9(66):110–8. 10.1098/rsif.2011.0182 . 21613286 PMC3223624

[55] Alexander N, Strutzenberger G, Ameshofer LM, Schwameder H. Lower limb joint work and joint work contribution during downhill and uphill walking at different inclinations. J Biomech. 2017;61:75–80. Epub 2017/07/25. 10.1016/j.jbiomech.2017.07.001 . 28734544

[56] Roberts TJ. The integrated function of muscles and tendons during locomotion. Comp Biochem Physiol A Mol Integr Physiol. 2002;133(4):1087–99. 10.1016/s1095-6433(02)00244-1 . 12485693

[57] Biewener AA, Roberts TJ. Muscle and tendon contributions to force, work, and elastic energy savings: a comparative perspective. Exerc Sport Sci Rev. 2000;28(3):99–107. . 10916700

[58] Roberts TJ, Azizi E. Flexible mechanisms: the diverse roles of biological springs in vertebrate movement. J Exp Biol. 2011;214(Pt 3):353–61. 10.1242/jeb.038588 . 21228194 PMC3020146

[59] Snyder KL, Kram R, Gottschall JS. The role of elastic energy storage and recovery in downhill and uphill running. Journal of Experimental Biology. 2012;215(13):2283–7. 22675189 10.1242/jeb.066332

[60] Shepertycky M, Li Q. Generating Electricity during Walking with a Lower Limb-Driven Energy Harvester: Targeting a Minimum User Effort. PLoS One. 2015;10(6):e0127635 10.1371/journal.pone.0127635 . 26039493 PMC4454656

[61] Cervera A, Rubinshtein Ze, Gad M, Riemer R, Peretz MM. Biomechanical Energy Harvesting System With Optimal Cost-of-Harvesting Tracking Algorithm. IEEE Journal of Emerging and Selected Topics in Power Electronics. 2016;4(1):293–302.

[62] MacLean MK, Ferris DP. Energetics of Walking With a Robotic Knee Exoskeleton. Journal of applied biomechanics. 2019;35(5):320–6. 10.1123/jab.2018-0384 31541067

[63] Sawicki GS, Beck ON, Kang I, Young AJ. The exoskeleton expansion: improving walking and running economy. Journal of NeuroEngineering and Rehabilitation. 2020;17(1):25 10.1186/s12984-020-00663-9 32075669 PMC7029455

[64] Kao PC, Lewis CL, Ferris DP. Invariant ankle moment patterns when walking with and without a robotic ankle exoskeleton. Journal of Biomechanics. 2010;43(2):203–9. 10.1016/j.jbiomech.2009.09.030 19878952 PMC2813403

[65] Lewis CL, Ferris DP. Invariant hip moment pattern while walking with a robotic hip exoskeleton. Journal of Biomechanics. 2011;44(5):789–93. 10.1016/j.jbiomech.2011.01.030 21333995 PMC3075111

[66] Koller JR, Remy CD, Ferris DP. Biomechanics and energetics of walking in powered ankle exoskeletons using myoelectric control versus mechanically intrinsic control. Journal of neuroengineering and rehabilitation. 2018;15(1):42 10.1186/s12984-018-0379-6 29801451 PMC5970476

[67] Mahon CE, Farris DJ, Sawicki GS, Lewek MD. Individual limb mechanical analysis of gait following stroke. Journal of Biomechanics. 2015;48(6):984–9. 10.1016/j.jbiomech.2015.02.006 25698237

[68] McCain EM, Dick TJM, Giest TN, Nuckols RW, Lewek MD, Saul KR, et al Mechanics and energetics of post-stroke walking aided by a powered ankle exoskeleton with speed-adaptive myoelectric control. J Neuroeng Rehabil. 2019;16(1):57 Epub 2019/05/17. 10.1186/s12984-019-0523-y . 31092269 PMC6521500

[69] Alexander RM. Optimum muscle design for oscillatory movements. Journal of theoretical Biology. 1997;184(3):253–9. 10.1006/jtbi.1996.0271 31940736

[70] Rubinshtein Ze, Peretz MM, Riemer R, editors. Biomechanical energy harvesting system with optimal cost-of-harvesting tracking algorithm. 2014 IEEE Applied Power Electronics Conference and Exposition-APEC 2014; 2014: IEEE.

[71] Xie L, Li X, Cai S, Huang G, Huang L. Knee-braced energy harvester: Reclaim energy and assist walking. Mechanical Systems and Signal Processing. 2019;127:172–89.

[72] Diller S, Majidi C, Collins SH, editors. A lightweight, low-power electroadhesive clutch and spring for exoskeleton actuation. 2016 IEEE International Conference on Robotics and Automation (ICRA); 2016: IEEE.

[73] Collins SH, Kuo AD. Controlled energy storage and return prosthesis reduces metabolic cost of walking. Power. 2005;600:800.

[74] Segal AD, Zelik KE, Klute GK, Morgenroth DC, Hahn ME, Orendurff MS, et al The effects of a controlled energy storage and return prototype prosthetic foot on transtibial amputee ambulation. Human movement science. 2012;31(4):918–31. 10.1016/j.humov.2011.08.005 22100728 PMC4302415

[75] Sutrisno A, Braun DJ. Enhancing mobility with quasi-passive variable stiffness exoskeletons. IEEE Transactions on Neural Systems and Rehabilitation Engineering. 2019;27(3):487–96. 10.1109/TNSRE.2019.2899753 30794186

[76] Montgomery JR, Grabowski AM. Use of a powered ankle–foot prosthesis reduces the metabolic cost of uphill walking and improves leg work symmetry in people with transtibial amputations. Journal of The Royal Society Interface. 2018;15(145):20180442. 10.1098/rsif.2018.0442 PMC6127176 30158189

