# Supplementary material for: Mechanics of walking and running up and downhill: A joint-level perspective to guide design of lower-limb exoskeletons
Source: PLoS One. 2020 Aug 28;15(8):e0231996. doi: 10.1371/journal.pone.0231996 (PMC7454943; doi:10.1371/journal.pone.0231996)
Supplement: S1 File — (DOCX) [file pone.0231996.s001.docx]

**Supplementary Metabolic Analysis**

**Title**

**Mechanics of walking and running up and downhill: A joint-level perspective to guide design of lower-limb exoskeletons**

**Authors**

Richard W. Nuckols

Kota Z. Takahashi

Dominic J. Farris

Sarai Mizrachi

Raziel Riemer

Gregory S. Sawicki

Metabolic Energy Expenditure for Walking and Running Up and Downhill

**Methods:** We collected and processed metabolic data for seven adults. Whole body metabolic energy expenditure was captured using a portable metabolic system (Oxycon Mobile, Viasys Healthcare, Yorba Linda, CA, USA). Rates of oxygen consumption and carbon dioxide production during trials were recorded and converted to metabolic powers using standard equations [1]. Baseline quiet standing metabolic rate was captured prior to gait trials. For each condition, respiratory data from minute 4 to 6 were averaged and used to report the steady state metabolic energy consumptions (watts) for the trial. The metabolic system reported values that were averaged over 30 second intervals so four values were averaged for each trial. In the most extreme case of 5.71º incline running, subjects could not complete the trial while maintaining a respiratory exchange ratio (RER) below one [2]. Therefore, only data from 3 out of 7 subjects are included for the 5.71º incline running condition. Task dependent metabolic power was calculated by subtracting the metabolic power in standing from the metabolic power recorded during the trial. These values were then normalized to each individual’s body mass. In addition, efficiency was calculated as the ratio of average total limb positive mechanical power to net metabolic power:

$\eta^{+}=\frac{P_{mech}^{+}}{P_{met}}$

where $\eta^{+}$ is efficiency of positive work, $P_{mech}^{+}$ is the average total limb positive power (summed across the lower-limb joints), and $P_{met}$ is mass normalized net metabolic power.

**Results:** In walking, the measured metabolic minimum was at -5.71º grade (1.5 W kg^-1^) (Supp Table 1). For running, the metabolic minimum was also at -5.71º grade (5.75 W kg^-1^) which was the steepest downhill grade tested in running. Efficiency of positive work was maximized at -5.71º grade in walking with an efficiency of 0.62.

**Supp Table 1:** Net metabolic power, summed (ankle +knee +hip) lower-limb joint average positive power, efficiency of positive joint work, and cost of transport for walking and running up and downhill.

|  | **Grade (deg)** | **P_MET_ (W kg^-1^)** | **P^+^ (W kg^-1^)** | **η^+^_WORK_** |
| --- | --- | --- | --- | --- |
| **Walk** | -8.53 | 2.24 | 0.86 | 0.38 |
| **(1.25 m s^-1^)** | -5.71 | 1.50 | 0.94 | 0.62 |
|  | 0 | 2.82 | 1.02 | 0.36 |
|  | 5.71 | 6.15 | 1.71 | 0.28 |
|  | 8.53 | 10.54 | 2.60 | 0.25 |
| **Run** | -5.71 | 5.75 | 2.64 | 0.46 |
| **(2.25 m s^-1^)** | -2.86 | 7.32 | 3.14 | 0.43 |
|  | 0 | 9.09 | 3.66 | 0.40 |
|  | 2.86 | 11.64 | 4.09 | 0.35 |
|  | 5.71 | 15.07 | 4.53 | 0.30 |

**Discussion:** Similar to Margaria *et al.* [3], we found that the greatest efficiency of positive work at -5.71º slope for both walking and running. Additionally, the efficiency of positive work during walking at the extreme uphill (+8.53º) was ~0.25 reflecting the efficiency of muscle-tendons during tasks exhibiting predominantly positive work [4-8].

**References:**

1. Brockway JM. Derivation of formulae used to calculate energy expenditure in man. Human nutritionClinical nutrition. 1987;41(6):463-71.

2. Farinatti P, NETO AGC, Amorim PR. Oxygen consumption and substrate utilization during and after resistance exercises performed with different muscle mass. International Journal of Exercise Science. 2016;9(1):77.

3. Margaria R, Cerretelli P, Aghemo P, Sassi G. Energy cost of running. J Appl Physiol. 1963;18:367-70. doi: 10.1152/jappl.1963.18.2.367. PubMed PMID: 13932993.

4. Margaria R. Positive and negative work performances and their efficiencies in human locomotion. Internationale Zeitschrift fur angewandte Physiologie, einschliesslich Arbeitsphysiologie. 1968;25(4):339-51. doi: 10.1007/bf00699624. PubMed PMID: 5658204.

5. Davies CT, Barnes C. Negative (eccentric) work. II. Physiological responses to walking uphill and downhill on a motor-driven treadmill. Ergonomics. 1972;15(2):121-31. doi: 10.1080/00140137208924416. PubMed PMID: 5036082.

6. Minetti AE, Moia C, Roi GS, Susta D, Ferretti G. Energy cost of walking and running at extreme uphill and downhill slopes. Journal of applied physiology (Bethesda, Md: 1985). 2002;93(3):1039-46. doi: 10.1152/japplphysiol.01177.2001 [doi].

7. Minetti AE, Ardigo LP, Saibene F. Mechanical determinants of gradient walking energetics in man. J Physiol. 1993;472:725-35. doi: 10.1113/jphysiol.1993.sp019969. PubMed PMID: 8145168; PubMed Central PMCID: PMCPMC1160509.

8. Zai CZ, Grabowski AM. The metabolic power required to support body weight and accelerate body mass changes during walking on uphill and downhill slopes. Journal of Biomechanics. 2020:109667.
